# Talking about Climate Change and Global Warming

**DOI:** 10.1371/journal.pone.0138996

**Published:** 2015-09-29

**Authors:** Maurice Lineman, Yuno Do, Ji Yoon Kim, Gea-Jae Joo

**Affiliations:** College of Natural Sciences, Department of Biological Sciences, Pusan National University, Busan, South Korea; Newcastle University, UNITED KINGDOM

## Abstract

The increasing prevalence of social networks provides researchers greater opportunities to evaluate and assess changes in public opinion and public sentiment towards issues of social consequence. Using trend and sentiment analysis is one method whereby researchers can identify changes in public perception that can be used to enhance the development of a social consciousness towards a specific public interest. The following study assessed Relative search volume (RSV) patterns for global warming (GW) and Climate change (CC) to determine public knowledge and awareness of these terms. In conjunction with this, the researchers looked at the sentiment connected to these terms in social media networks. It was found that there was a relationship between the awareness of the information and the amount of publicity generated around the terminology. Furthermore, the primary driver for the increase in awareness was an increase in publicity in either a positive or a negative light. Sentiment analysis further confirmed that the primary emotive connections to the words were derived from the original context in which the word was framed. Thus having awareness or knowledge of a topic is strongly related to its public exposure in the media, and the emotional context of this relationship is dependent on the context in which the relationship was originally established. This has value in fields like conservation, law enforcement, or other fields where the practice can and often does have two very strong emotive responses based on the context of the problems being examined.

## Introduction

Identifying trends in the population, used to be a long and drawn out process utilizing surveys and polls and then collating the data to determine what is currently most popular with the population [1, 2]. This is true for everything that was of merit to the political organizations present, regarding any issue of political or public interest.

Recently, the use of the two terms ‘Climate Change’ and ‘Global Warming’ have become very visible to the public and their understanding of what is happening with respect to the climate [3]. The public response to all of the news and publicity about climate has been a search for understanding and comprehension, leading to support or disbelief. The two terms while having similarity in meaning are used in slightly different semantic contexts. The press in order to expand their news readership/viewer lists has chosen to use this ambiguity to their favor in providing news to the public [4]. Within the news releases, the expression ‘due to climate change’ has been used to explain phenomological causality.

These two terms “global warming–(GW)” and “climate change–(CC)” both play a role in how the public at large views the natural world and the changes occurring in it. They are used interactively by the news agencies, without a thought towards their actual meaning [3, 4]. Therefore, the public in trying to identify changes in the news and their understanding of those changes looks for the meaning of those terms online. The extent of their knowledge can be examined by assessing the use of the terms in online search queries. Information searches using the internet are increasing, and therefore can indicate public or individual interest.

Internet search queries can be tracked using a variety of analytic engines that are independent of, or embedded into, the respective search engines (google trend, naver analytics) and are used to determine the popularity of a topic in terms of internet searches [5]. The trend engines will look for selected keywords from searches, keywords chosen for their relevance to the field or the query being performed.

The process of using social media to obtain information on public opinion is a practice that has been utilized with increasing frequency in modern research for subjects ranging from politics [6, 7] to linguistics [8–10] complex systems [11, 12] to environment [13]. This variety of research belies the flexibility of the approach, the large availability of data availability for mining in order to formulate a response to public opinion regarding the subject being assessed. In modern society understanding how the public responds regarding complex issues of societal importance [12].

While the two causally connected terms GW and CC are used interchangeably, they describe entirely different physical phenomena [14]. These two terms therefore can be used to determine how people understand the parallel concepts, especially if they are used as internet search query terms in trend analysis. However, searching the internet falls into two patterns, searches for work or for personal interest, neither of which can be determined from the trend engines. The By following the searches, it is possible to determine the range of public interest in the two terms, based on the respective volumes of the search queries. Previously in order to mine public opinion on a subject, government agencies had to revert to polling and surveys, which while being effective did not cover a very large component of the population [15–17].

Google trend data is one method of measuring popularity of a subject within the population. Individuals searching for a topic use search keywords to obtain the desired information [5, 18]. These keywords are topic sensitive, and therefore indicate the level of knowledge regarding the searched topic. The two primary word phrases here “climate change” and “global warming” are unilateral terms that indicate a level of awareness about the issue which is indicative of the individuals interest in that subject [5, 19, 20]. Google trend data relates how often a term is searched, that is the frequency of a search term can be identified from the results of the Google® trend analysis. While frequency is not a direct measure of popularity, it does indicate if a search term is common or uncommon and the value of that term to the public at large. The relationship between frequency and popularity lies in the volume of searches by a large number of individuals over specific time duration. Therefore, by identifying the number of searches during a specific period, it is possible to come to a proximate understanding of how popular or common a term is for the general population [21]. However, the use of trend data is more appropriately used to identify awareness of an issue rather than its popularity.

This brings us to sentiment analysis. Part of the connection between the search and the populations’ awareness of an issue can be measured using how they refer to the subject in question. This sentiment, is found in different forms of social media, or social networking sites sites i.e. twitter®, Facebook®, linked in® and personal blogs [7, 22–24]. Thus, the original information, which was found on the internet, becomes influenced by personal attitudes and opinions [25]and then redistributed throughout the internet, accessible to anyone who has an internet connection and the desire to search. This behavior affects the information that now provides the opportunity to assess public sentiment regarding the prevailing attitudes regarding environmental issues [26, 27]. To assess this we used Google® and Twitter® data to understand public concerns related to climate change and global warming. Google trend was used to trace changes in interest between the two phenomena. Tweets (comments made on Twitter®) were analyzed to identify negative or positive emotional responses.

Comparatively, twitter data is more indicative of how people refer to topics of interest [28–31], in a manner that is very linguistically restricted. As well, twitter is used as a platform for verbal expression of emotional responses. Due to the restrictions on tweet size (each tweet can only be 140 characters in length), it is necessary to be more direct in dealing with topics of interest to the tweeter. Therefore, the tweets are linguistically more emotionally charged and can be used to define a level of emotional response by the tweeter.

The choice of target words for the tweets and for the Google trend searches were the specific topic phrases [32, 33]. These were chosen because of the descriptive nature of the phrases. Scientific literature is very specific in its use and therefore has very definitive meanings. The appropriation of these words by the population as a method for describing their response to the variation in the environment provides the basis for the choice as target words for the study. The classification of the words as being positive versus negative lies in the direction provided by Frank Lutz. This politicization of a scientific word as a means of directing public awareness, means the prescription of one phrase (climate change) as being more positive than the other (global warming).

Global warming is defined as the long-term trend of increasing average global temperatures; alternatively, climate change is defined as a change in global or regional climate patterns, in particular a change apparent from the mid to late 20^th^ century onwards and attributed to the increased levels of atmospheric carbon dioxide arising from the use of fossil fuels. Therefore, the search keywords were chosen based on their scientific value and their public visibility. What is important about the choice of these search terms is that due to their scientific use, they describe a distinctly identifiable state. The more specific these words are, the less risk of the algorithm misinterpreting the keyword and thus having the results misinterpreted [34–36].

The purpose of the following study was to identify trends within search parameters for two specific sets of trend queries. The second purpose of the study was to identify how the public responds emotionally to those same queries. Finally, the purpose of the study was to determine if the two had any connections.

## Methods

### Data Collection

Public awareness of the terms climate change and global warming was identified using Google Trends (google.com/trends) and public databases of Google queries [37]. To specify the exact searches we used the two terms ‘climate change’ and ‘global warming’ as query phrases. Queries were normalized using relative search volume (RSV) to the period with the highest proportion of searches going to the focal terms (i.e. RSV = 100 is the period with the highest proportion for queries within a category and RSV = 50 when 50% of that is the highest search proportion). Two assumptions were necessary for this study. The first is, of the two terms, climate change and global warming, that which draws more search results is considered more interesting to the general population. The second assumption is that changes in keyword search patterns are indicators of the use of different forms of terminology used by the public. To analyze sentiments related to climate change and global warming, tweets containing acronyms for climate change and global warming were collected from Twitter API for the period from October 12 to December 12, 2013. A total of 21,182 and 26,462 tweets referencing the terms climate change and global warming were collected respectively. When duplicated tweets were identified, they were removed from the analysis. The remaining tweets totaled 8,465 (climate change) and 8,263 (global warming) were compiled for the sentiment analysis.

### Data Analysis

In Twitter® comments are emotionally loaded, due to their textually shortened nature. Sentiment analysis, which is in effect opinion mining, is how opinions in texts are assessed, along with how they are expressed in terms of positive, neutral or negative content [36]. Nasukawa and Yi [10]state that sentiment analysis identifies statements of sentiment and classifies those statements based on their polarity and strength along with their relationship to the topic.

Sentiment analysis was conducted using Semantria® software (www.semantria.com), which is available as an MS Excel spreadsheet application plugin. The plugin is broken into parts of speech (POS), the algorithm within the plugin then identifies sentiment-laden phrases and then scores them from -10 to 10 on a logarithmic scale, and finally the scores for each POS are tabulated to identify the final score for each phrase. The tweets are then via statistical inferences tagged with a numerical value from -2 to 2 and given a polarity, which is classified as positive, neutral or negative [36]. Semantria®, the program utilized for this study, has been used since 2011 to perform sentiment analyses [7, 22].

For the analysis, an identity column was added to the dataset to enable analysis of individual tweets with respect to sentiment. A basic sentiment analysis was conducted on the dataset using the Semantria® plugin. The plugin uses a cloud based corpus of words tagged with sentimental connotations to analyze the dataset. Through statistical inference, each tweet is tagged with a sentiment value from -2 to +2 and a polarity of (i) negative, (ii) neutral, or (iii) positive. Positive nature increases with increasing positive sentiment. The nature of the language POS assignation is dependent upon the algorithmic classification parameters defined by the Semantria® program. Determining polarity for each POS is achieved using the relationship between the words as well as the words themselves. By assigning negative values to specific negative phrases, it limits the use of non-specific negation processes in language; however, the program has been trained to assess non-specific linguistic negations in context.

A tweet term frequency dictionary was computed using the N-gram method from the corpus of climate change and global warming [38]. We used a combination of unigrams and bigrams, which has been reported to be effective [39]. Before using the N-gram method, typological symbols were removed using the open source code editor (i.e. Notepad) or Microsoft Words’ “Replace” function.

Differences in RSV’s for the terms global warming and climate change for the investigation period were identified using a paired t-test. Pettitt and Mann-Kendall tests were used to identify changes in distribution, averages and the presence of trends within the weekly RSV’s. The Pettitt and MK tests, which assume a stepwise shift in the mean (a break point) and are sensitive to breaks in the middle of a time series, were applied to test for homogeneity in the data [40]. Temporal trends within the time series were analyzed with Spearman’s non-parametric correlation analysis. A paired t-test and Spearman’s non-parametric correlation analysis were conducted using SPSS software (version 17.0 SPSS In corp. Chicago IL) and Pettitt and MK tests were conducted using XLSTAT (version 7.0).

To determine the accuracy and reliability of the Sentiment analysis, a Pearson’s chi-square analysis was performed. This test identifies the difference ratio for each emotional response group, and then compares them to determine reliance and probability of interactions between the variables, in this case the terms global warming and climate change.

## Results

**Fig 1:**
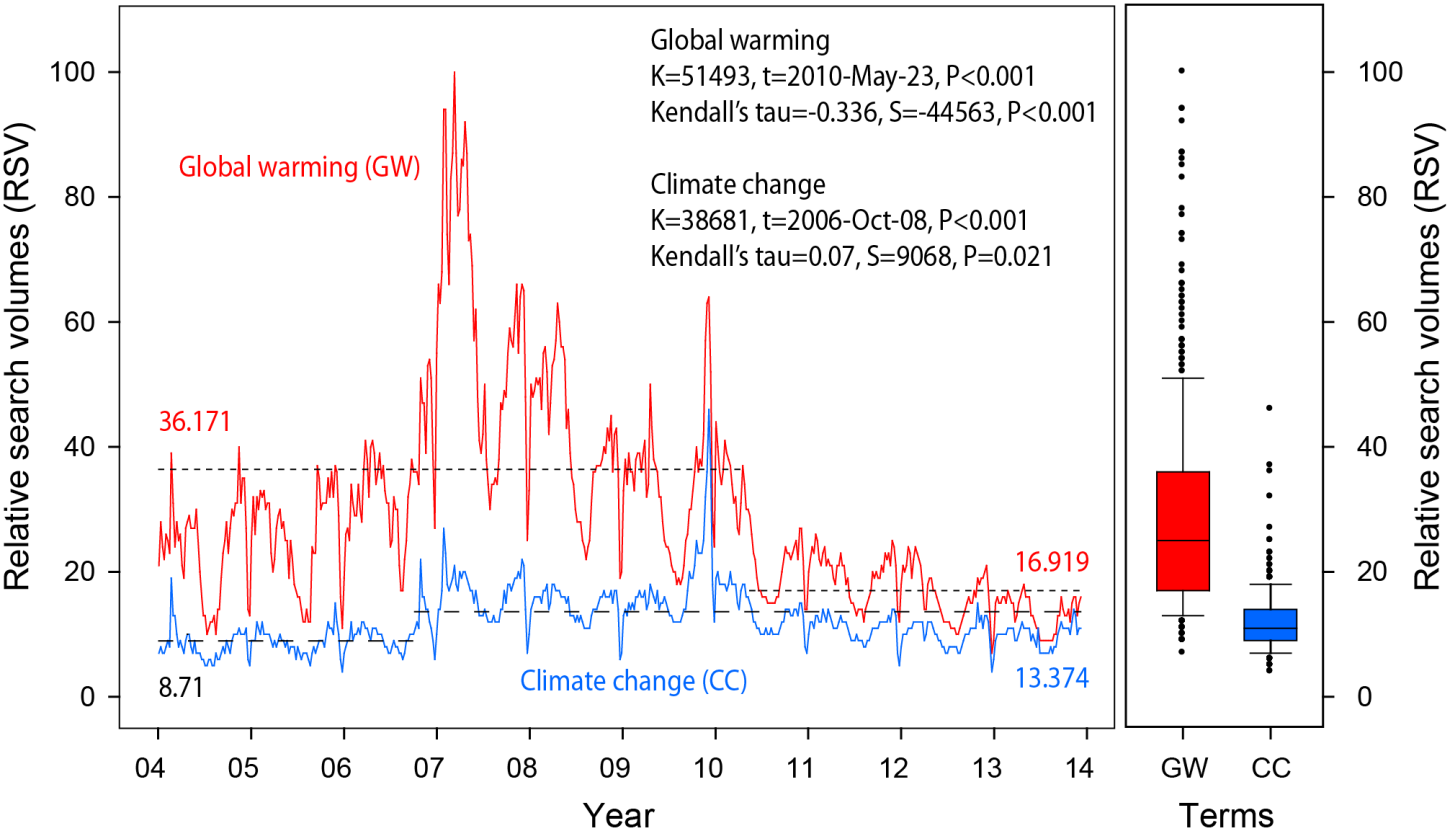
Change in relative search volume (RSV) for “global warming” and “climate change” as search terms (2007–2013); dash line represents the mean for the RSV for each period.

According to Google trend (Fig 1) from 2004–2014, people searched for the term global warming (n = 8,464; mean ± S.D = 25.33 ± 2.05) more frequently than climate change (n = 8,283; mean ± S.D. = 7.97±0.74). Although the Intergovernmental Panel on Climate Change (IPCC) published its Fourth Assessment Report in 2007 and was awarded the Nobel Prize, interest in the term global warming as used in internet searches has decreased significantly since 2010 (K = 51493, t = 2010-May-23, P<0.001). Further the change in RSV also been indicative of the decreased pattern (Kendall’s tau = -0.336, S = -44563, P<0.001). The use of the term “climate change” has risen marginally since 2006 (K = 38681, t = 2006-Oct-08, P<0.001), as indicated by a slight increase (Kendall’s tau = -0.07, S = 9068, P<0.001). These findings show that the difference in usage of the two terms climate change and global warming has recently been reduced.

**Fig 2:**
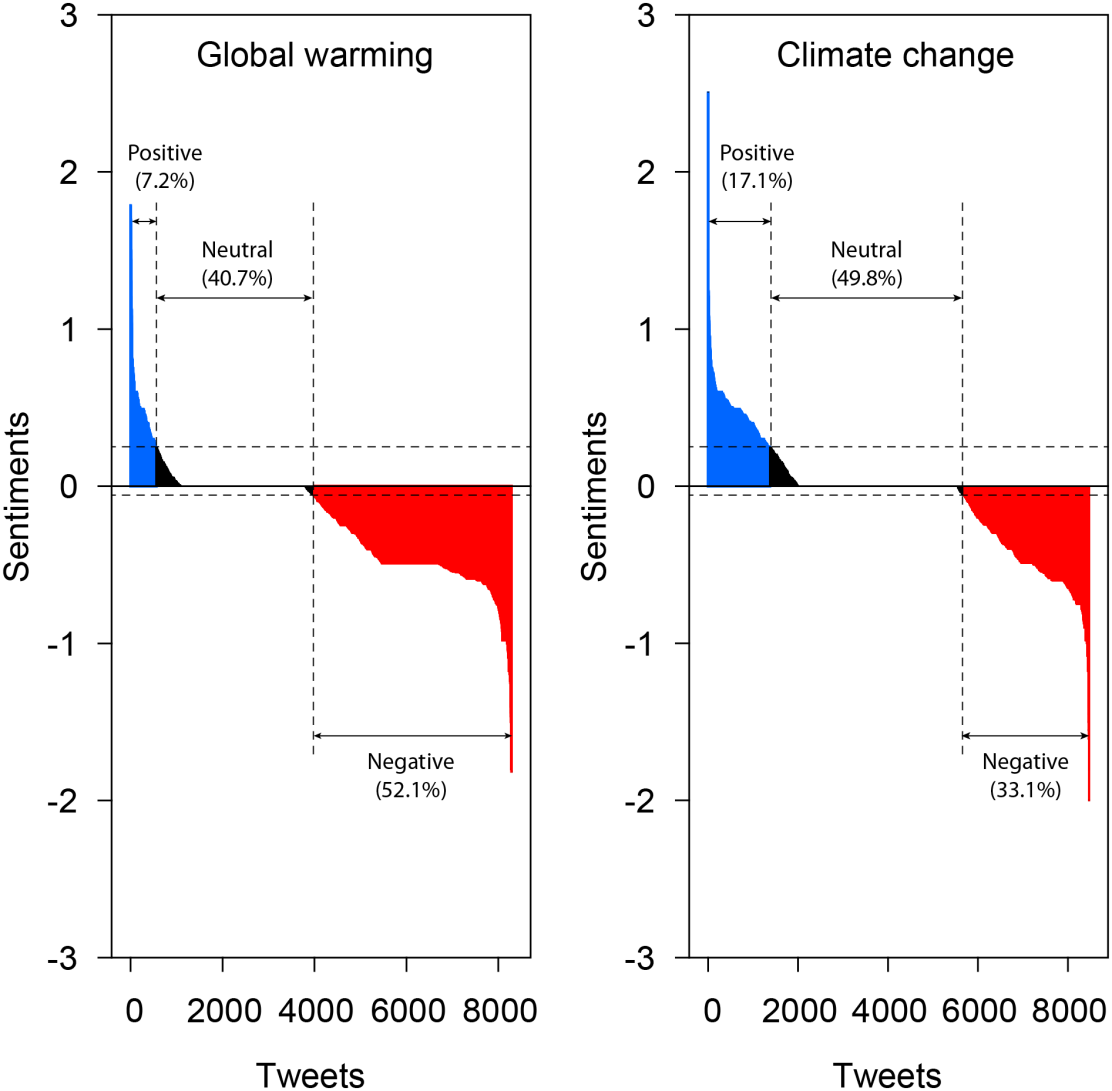
Distribution of positive, neutral, and negative sentiments in tweets about global warming and climate change.

The sentiment analysis of tweets (Fig 2) shows that people felt more negative about the term global warming (sentiment index = -0.21±0.34) than climate change (-0.068±0.36). Global warming tweets reflecting negative sentiments via descriptions such as, “bad, fail, crazy, afraid and catastrophe,” represented 52.1% of the total number of tweets. As an example, the tweet, “Supposed to snow here in the a.m.! OMG. So sick of already, but Saturday says 57 WTF!” had the lowest score at -1.8. Another observation was that 40.7% of tweets, including “agree, recommend, rescue, hope, and contribute,” were regarded as neutral. While 7.2% of tweets conveyed positive messages such as, “good, accept, interesting, and truth.” One positive global warming tweet, read, “So if we didn’t have global warming, would all this rain be snow!”. The results from the Pearson’s chi-square analysis showed that the relationship between the variables was significant (Pearson’s chi-square –763.98, d.f. = 2, P<0.001). Negative climate change tweets represented 33.1% of the total while neutral tweets totaled 49.8%, while positive climate change tweets totaled 17.1%.

Understandably, global warming and climate change are the terms used most frequently to describe each phenomenon, respectively, as revealed by the N-gram analysis (Table 1). When people tweeted about global warming, they repeatedly used associated such as, “ice, snow, Arctic, and sea.” In contrast, tweets referring to climate change commonly used, “report, IPCC, world, science, environment, and scientist.” People seem to think that climate change as a phenomenon is revealed by scientific investigation.

**Table 1:** Tweet Terms-frequency Dictionary for Global Warming and Climate Change.

Rank	Global warming		Climate change	
	Words	Frequency	Words	Frequency
1	Global warming	821	Climate change	802
2	Climate change	507	Global warming	267
3	Ice	177	Ow	177
4	Years	158	Report	167
5	Snow	143	IPCC	143
6	Arctic	136	World	136
7	Scientist	124	Science	119
8	Sea	119	Environment	105
9	Cause	114	Scientist	101
10	Ow	109	Help	100
11	Time	101	Action	97
12	Show	97	Impacts	85
13	Report	94	Arctic	82
14	Science	91	Time	79
15	Data	88	Australia	77
16	World	85	Study	75
17	Earth	82	Caused	72
18	Environment	75	Talk	70
19	Coverage	70	Human	68
20	Percent	68	Need	65
21	Human	67	People	63
22	Study	65	Deniers	60
23	Satellite	63	Huff	58
24	IPCC	60	Risk	57
25	EPA	56	Fight	56
26	Expert	54	Years	54
27	Stop	53	Make	53
28	Fight	52	Politics	52
29	Million	51	Nations	51
30	People	50	Carbon	49

## Discussion

Internet searches are one way of understanding the popularity of an idea or meme within the public at large. Within that frame of reference, the public looks at these two terms global warming and climate change and their awareness of the roles of the two phenomena [41]. From 2004 to 2008, the search volumes for the term global warming far exceeded the term climate change. The range for the term global warming in Relative search volumes (RSV) was more than double that of climate change in this period (Fig 1). From 2008 on the RSV’s began to steadily decrease until in 2014 when the RSV’s for the term global warming were nearly identical to those for the term climate change. From 2008 there was an increase in the RSVs for CC until 2010 at which point the RSVs also began to decline for the term climate change. The decline in the term climate change for the most part paralleled that of the term global warming from 2010 on to the present.

While we are seeing the increases and decreases in RSVs for both the terms global warming and climate change, the most notable changes occur when the gap between the terms was the greatest, from 2008 through to 2010. During this period, there was a very large gap found between the RSVs for the terms global warming and climate change; however, searches for the term climate change was increasing while searches for the tem global warming were decreasing. The counter movement of the RSV’s for the two terms shows that there is a trend happening with respect to term recognition. At this point, there was an increase in the use of the CC term while there was a corresponding decrease in the use of the GW term. The change in the use of the term could have been due to changes in the publicity of the respective terms, since at this point, the CC term was being used more visibly in the media, and therefore the CC term was showing up in headlines and the press, resulting in a larger number of searches for the CC term. Correspondingly, the decrease in the use of the GW term is likely due to the changes in how the term was perceived by the public. The public press determines how a term is used, since they are the body that consistently utilizes a term throughout its visible life. The two terms, regardless of how they differ in meaning, are used with purpose in a scientific context, yet the public at large lacks this definition and therefore has no knowledge of the variations in the terms themselves [42]. Therefore, when searching for a term, the public may very well, choose the search term that they are more comfortable with, resulting in a search bias, since they do not know the scientific use of the term.

The increase in the use of the CC term, could be a direct result of the release of the fourth assessment report for the IPCC in 2007 [43]. The publicity related to the release of this document, which was preceded by the release of the Al Gore produced documentary “An Inconvenient Truth”, both of which were followed by the selection by the Nobel committee of Al Gore and the IPCC scientists for the Nobel Prize in 2007 [43]. These three acts individually may not have created the increased media presence of the CC term; however, at the time the three events pushed the CC term and increased its exposure to the public which further drove the public to push for positive environmental change at the political level [44, 45]. This could very well have resulted in the increases in RSV’s for the CC term. This point is more likely to depict accurately the situation, since in 2010 the use of the two terms decline at almost the same rate, with nearly the same patterns.

Thus with respect to trend analysis, what is interesting is that RSVs are paralleling the press for specific environmental events that have predetermined value according to the press. The press in increasing the visibility of the term may drive the increases in the RSV’s for that term. Prior to 2007, the press was using the GW term indiscriminately whenever issues affecting the global climate arose; however, after the movie, the report and then the Nobel prize the terminology used by the press switched and the CC term became the word du jour. This increased the visibility of the word to the public, thereby it may be that increasing public awareness of the word, but not necessarily its import, is the source for the increases in RSV’s between 2008 and 2010.

The decline in the RSV’s then is a product of the lack of publicity about the issue. As the terms become more familiar, there would be less necessity to drive the term publicly into the spotlight; however, occasionally events/situations arise that refocus the issue creating a resurgence in the terms even though they have reached their peak visibility between 2008 and 2010.

Since these terms have such an impact on the daily lives of the public via local regional national and global weather it is understandable that they have an emotional component to them [46]. Every country has its jokes about the weather, where they come up with cliché’s about the weather (i.e. if you don’t like the weather wait 10minutes) that often show their discord and disjunction with natural climatological patterns [47]. Furthermore, some sectors of society (farmers) have a direct relationship with the climate and their means of living; bad weather is equal to bad harvests, which means less money. To understand how society represents this love hate relationship with the weather, the twitter analysis was performed. Twitter, a data restricted social network system, has a limited character count to relay information about any topic the sender chooses to relate. These tweets can be used to assess the sentiment of the sender towards a certain topic. As stated previously, the sentiment is defined by the language of the tweet within the twitter system. Sentiment analysis showed that the two terms differed greatly. Based on the predefined algorithm for the sentiment analysis, certain language components carried a positive sentiment, while others carried a negative sentiment. Tweets about GW and CC were subdivided based on their positive, neutral and negative connotations within the tweet network. These emotions regardless of their character still play a role in how humans interacts with surroundings including other humans [48, 49] As seen in Fig 2 the different terms had similar distributions, although with different ranges in the values. Global warming showed a much smaller positive tweet value than did climate change. Correspondent to this the respective percentage of positive sentiments for CC was more than double that of GW. Comparatively, the neutral percentiles were more similar for each term with a small difference. However, the negative sentiments for the two terms again showed a greater disparity, with negative statements about GW nearly double those of climate change.

These differences show that there is a perceptive difference in how the public relates to the two terms Global Warming and Climate Change [50, 51]. Climate change is shown in a more positive light than global warming simply based on the tweets produced by the public. The difference in how people perceive climate change and global warming is possibly due to the press, personal understanding of the terms, or level of education. While this in itself is indefinable, since by nature tweets are linguistically restrictive, the thing to take from it is that there is a measurable difference in how individuals respond to climatological changes that they are experiencing daily. These changes have a describable effect on how the population is responding to the publicity surrounding the two terms to the point where it can be used to manipulate governmental policy [52].

Sentiment analysis is a tool that can be used to determine how the population feels about a topic; however, the nature of the algorithm makes it hard to effectively determine how this is being assessed. For the current study, the sentiment analysis showed that there was a greater negative association with the term global warming than with the term climate change. This difference, which while being an expression of individual like or dislike at the time the tweet was created, denotes that the two terms were either not understood in their true form, or that individuals may have a greater familiarity with one term over the other, which may be due to a longer exposure to the term (GW) or the negative press associated with the term (GW).

## Conclusions

Trend analysis identified that the public is aware of the terminology used to describe climatological variation. The terminology showed changes in use over time with global warming starting as the more well-known term, and then its use decreased over time. At the same time, the more definitive term climate change had less exposure early on; however, with the increase of press exposure, the public became increasingly aware of the term and its more accurate definition. This increase appeared to be correspondent with the increasing publicity around three very powerful press exposure events (a documentary, a scientific report release and a Nobel Prize). The more the term was used the more people came to use it, this included searches on the internet.

Comparatively sentiment analysis showed that the two terms had differential expressions in the population. With climate change being seen in a more positive frame than global warming. The use of sentiment analysis as a tool to evaluate how the population is responding to a feature is an important tool. However, it is a tool that measures, it does not define.

Social network systems and internet searches are effective tools in identifying changes in both public awareness and public perception of an issue. However, in and of itself, these are bell ringers they can be used to determine the importance of an issue, but not the rationale behind the why it is important. This is an important fact to remember when using analytical tools that evaluate social network systems and their use by the public.
